# Are You Qualified, ChatGPT? Examining Clinical Skills and Competencies of ChatGPT in Delivering Systemic Interventions

**DOI:** 10.1111/jmft.70168

**Published:** 2026-08-31

**Authors:** Gizem Erdem, Nilüfer Kafescioğlu, Gökçenay Başer

**Affiliations:** ^1^ Department of Psychology Koç University Istanbul Türkiye; ^2^ Department of Psychology Istanbul Bilgi University Istanbul Türkiye; ^3^ Department of Educational Psychology Texas A&M University College Station Texas USA

**Keywords:** AI therapy, artificial intelligence, common factors, cultural competence, generative AI, systemic therapy, therapist competence

## Abstract

Large language model agents such as ChatGPT are increasingly used for relationship advice and mental health support, with many users describing these interactions as therapeutic. This study assessed the clinical and cultural competence of a GPT‐based model trained to provide systemic interventions aligned with the AAMFT Code of Ethics and core skills. A volunteer client discussed a relational concern with the model, and the resulting transcript was evaluated by 12 experienced supervisors using standardized measures. Supervisors, unaware of the study's purpose and the therapist being an AI, rated the model as moderately competent in maintaining a systemic perspective and delivering systemic interventions. Supervisors identified weaknesses in pacing, joining, and attending to cultural and contextual factors, and inferred that the therapist resembled an early‐career clinician with less than 500 h of experience. Findings indicate limitations of AI therapy and underscore the value of human judgment, attunement, and intuition in clinical practice.

## 1 Introduction

Large Language Model (LLM) based generative Artificial Intelligence (AI) agents such as ChatGPT, Gemini, and Deep Seek have increasingly been popular with use patterns varying from writing, reviewing, summarizing, and translation of text to more complex tasks for organization, research, and entertainment (Chatterji et al. 2025; Maslej et al. 2025). One emerging (and rather controversial) use pattern relates to using AI agents to seek relational advice and therapeutic support for mental health issues. In a nationally representative survey in the United States, 13.1% of youth (12–21 years of age) reported using generative AI for mental health advice (McBain et al. 2025). Studies with clinical populations reveal a higher prevalence of AI use, suggesting that 48.7% of adult clients (18‐80 years of age) reported utilizing LLMs for therapeutic support (Rousmaniere et al. 2025). Research examining the ChatGPT conversation data indicates that while the most common topics of interactions were practical guidance, writing, and seeking information (78% of all messages), advice seeking and asking for guidance were notably high, accounting for 49% of messages (Chatterji et al. 2025).

Given the growing public interest and demand in using AI for mental health support, there has been a proliferation of commercial AI therapy apps that deliver evidence‐based mental health interventions, and they have a large user base. For instance, the leading AI therapy app, Wysa, has approximately 4.5 million users across +65 countries with established numerous partnerships with employers across the globe (Baldry 2022). Studies document that users report high satisfaction with the AI therapy apps, build a therapeutic alliance with their AI therapist over time (Beatty et al. 2022), and perceive them as nonjudgmental, non‐labeling, while enjoying their constant availability at low cost (Chaudhry and Debi 2024).

Despite positive reviews of AI therapy app users and advocates of AI use in psychotherapy^1^ underscoring their accessibility, affordability, and immediacy, research examining outcomes of AI use for mental health tells a different story. Meta‐analyses examining the efficacy of AI therapy apps across general mental health problems (He et al. 2023) as well as specific symptoms such as depression and anxiety (Zhong et al. 2024) have found mixed findings with effect sizes ranging from 0.19 to 0.62. While AI‐based psychotherapy provide rapid symptom relief (Balan et al. 2024; Li et al. 2023), the gains are not maintained in the long term (Zhong et al. 2024). As such, there is a need for further research to explore the quality, rather than subjective evaluation of AI agents and the direct and indirect outcomes of AI agent use for therapeutic purposes. Furthermore, there are ongoing concerns over confidentiality, data privacy, risks to the client in case of intensive clinical needs, and limited cultural competency (Maslej et al. 2025; McBain et al. 2025; Rahsepar Meadi et al. 2025).

The current study aims to address these gaps in the literature by exploring clinical supervisors’ evaluations of skills and competencies of a trained GPT model from a systemic perspective. The emphasis on systemic orientation in this study is also purposeful. The majority of the existing literature exploring AI agent use for therapeutic purposes has focused on manualized and protocol‐driven approaches, such as cognitive‐behavioral therapy models, given the ease of training AI agents on them due to their standardized nature (He et al. 2023; Li et al. 2023; Zhong et al. 2024). Nevertheless, recent reports show that many users predominantly turn to AI for relational advice (American Psychological Association 2025), making it increasingly important to examine how well AI models can deliver interventions oriented toward couple and family dynamics, which is an area that has received little research attention.

### 1.1 Human‐AI Therapy Process

The technical advancements of conversational AI systems have enabled LLMs to generate responses that closely resemble therapeutic processes, including empathic reflection, normalization, and emotional validation (Scholich et al. 2025). LLMs have shown the ability to recognize emotional states from text, voice, and images, demonstrating a form of cognitive empathy (Başer et al. 2025; Yirmiya and Fonagy 2025). Empirical findings indicate that users interacting with ChatGPT for emotional and mental health support report feeling emotionally connected and attribute therapist‐like qualities to the AI, such as professionalism, reliability, and a caring, validating presence (Luo et al. 2025). In some cases, recipients have reported that AI‐generated responses were more supportive than those from non‐professional human responses (e.g., Elyoseph et al. 2023; Yin et al. 2024). These findings suggest that AI‐generated therapeutic dialogue can be perceived as emotionally attuned and supportive.

Despite these capabilities, the perceived emotional capacity and connection demonstrated by AI differ from those of human connections. AI‐generated empathy is referred to as *pseudo‐empathy*, which lacks experiential understanding, relational commitment, emotional insight, and thus authentic affective empathy (Yirmiya and Fonagy 2025). This distinction renders AI systems incapable of relational reciprocity, countertransference, and embodied attunement, all of which characterize effective therapeutic relationships (Yirmiya and Fonagy 2025). Moreover, psychotherapy is theoretically diverse and deeply relational, making therapeutic competence difficult to define or standardize (Grodniewicz and Hohol 2023). These complexities are particularly important for non‐human agents, as they might mimic the relational and culturally responsive dimensions of therapy linguistically without fully comprehending their meaning and associated attributes.

Users’ perceived competence of AI agents may mask their fundamental limitations, which raises ethical concerns about overestimating AI's therapeutic capacity (Barzkar et al. 2025). When users cannot differentiate between AI‐generated support and genuine psychotherapy, they become vulnerable to “therapeutic misconception” (Khawaja and Bélisle‐Pipon 2023). This risk is particularly concerning for vulnerable populations, such as trauma survivors, as AI simulations could carry the risk of creating harmful relational dynamics (Yirmiya and Fonagy 2025). AI's ability to mimic therapeutic language may create a false sense of alliance and care (Khawaja and Bélisle‐Pipon 2023), highlighting the need for professional oversight and systematic evaluation. Detecting these risks through user perceptions alone is challenging and highlights the importance of examining how trained clinicians evaluate AI‐generated therapeutic responses, particularly regarding clinical skill, ethical boundaries, and cultural responsiveness. Without such evaluations, it becomes difficult to distinguish between AI applications that genuinely support mental health and those that create a sense of care while potentially carrying risks.

Lastly, cultural competence remains as a critical limitation of AI models in therapeutic contexts (Wang et al. 2025). Clinical evaluations reveal that LLMs often offer oversimplified advice regardless of specific cultural background, lived experiences, or situational realities, reflecting an important deficit in contextual adaptation (Iftikhar et al. 2025). Users and researchers alike have reported on such contextualization problems where AI therapy apps (i.e., Woebot, Wysa) and LLM‐based chatbots (i.e., ChatGPT) were unable to understand and interpret the human context in which the messages were embedded and instead provided generic, predictable, and repetitive responses (Chaudhry and Debi 2024; Sarkar et al. 2023). Taken together, these limitations suggest that examining the qualifications of AI agents in therapeutic contexts also requires assessment of their cultural and contextual responsiveness.

### 1.2 AI Use for Relational Therapy

The literature identifies four key areas in which AI holds potential for use in relational therapy: diagnosing relationship problems, predicting prognosis, monitoring client reactions, and providing treatment (Puhlman and Chen 2025). The present study focuses specifically on the last area, the provision of treatment. A growing body of evidence supports the effectiveness of digital relationship interventions more broadly (Kernová et al. 2025), including well‐established web‐based programs such as OurRelationship (Doss et al. 2020). However, a critical limitation of these programs is their dependence on human coaching to sustain engagement: completion rates fall to 6% when there is no coaching involved, with the benefit of coaching attributed to accountability and encouragement rather than clinical skill (Knopp et al. 2023). Thus, researchers have proposed AI chatbots and AI therapy apps as a solution to provide such interactions without consuming human resources (Knopp et al. 2023).

Nonetheless, research on the use of AI specifically for relational therapy is still in its infancy. A noteworthy exception is a recent randomized controlled trial by Vowels et al. (2025), in which adults with specific relationship issues were assigned to either a single session with a chatbot or an evidence‐based conflict reappraisal writing task. Results indicated similar improvements in both groups across various domains such as relationship satisfaction, dyadic coping, problem‐specific confidence, and individual well‐being (Vowels et al. 2025). One significant between‐group difference was found in the short‐term improvement of the demand‐withdraw communication pattern specifically for the chatbot condition, although this effect disappeared at follow‐up. While the participants rated the chatbot highly on therapeutic alliance, it was also described as robotic, repetitive, and only moderately human‐like. Additionally, the researchers raised safety concerns, noting that of the three conversations that warranted a safety follow‐up, the chatbot initiated one.

These limitations reflect broader challenges in applying AI to relational contexts. Puhlman and Chen (2025) identified three interconnected challenges. First, there is the difficulty of accessing sufficient conversation data from couples and family therapy contexts, which are necessary for AI training. Second, due to the complexity and dynamic nature of therapeutic strategies, it is difficult to integrate clinical knowledge into AI training. Third, it is not possible to explain why a model produced a particular response, making it difficult to evaluate whether the output was clinically appropriate or ethically sound. Thus, while AI holds promise for relational therapy, the technology is not yet sufficiently developed to function reliably in this context. In the context of couples and families, these gaps risk amplifying miscommunication or escalating conflict rather than alleviating it (Puhlman and Chen 2025).

Beyond the technical and clinical limitations described above, AI's integration into relational therapy poses significant questions about the future of the field of Couple and Family Therapy (CFT). According to Hertlein and Springer (2026), AI is already built into many telehealth platforms and software systems used by CFTs, making AI literacy essential for effective and ethical practice. They argue that AI has evolved beyond being merely a tool and now acts as a third participant within the therapeutic system. This evolution requires the CFTs to consider how to incorporate AI while preserving their systemic and relational approaches and treatment objectives. Moreover, the authors emphasize that if CFTs do not proactively engage with AI and guide its application in relational contexts, its use may not align with the relational and systemic standards of our field. Accordingly, the authors call for the development of field‐specific regulatory standards and for empirical research to guide the responsible use of AI in relational therapy.

### 1.3 The Current Study

In an attempt to address those limitations in the literature, the goal of the current study is to examine AI‐human interactions during a mock session and assess the quality of AI‐generated micro‐interventions through evaluations of experienced CFT clinical supervisors. To that aim, supervisors (who were blinded to the study) reviewed the mock session transcript and rated therapeutic skills, competence, and cultural responsiveness of the so‐called “therapist.” The research questions were as follows: (1) What are the most frequently used skills and micro‐interventions that a systemically trained AI agent uses while addressing a relational issue? (fidelity to systemic therapy principles). (2) According to the clinical supervisors, how competent is the AI agent to deliver systemic interventions? (adherence: clinical competence). (3) According to the clinical supervisors, how competent is AI to deliver micro‐interventions with cultural sensitivity and awareness? (cultural competence). (4) What are the strengths and areas of growth that clinical supervisors identify for the AI agent?

We hypothesized that the GPT (an AI agent the research team trained in CFT theories, skills, and interventions) would demonstrate high fidelity to systemic principles (H1) and high competence in basic interviewing skills, such as validating, mirroring, and summarizing (H_2a_). We expected that GPT would perform poorly in more complex clinical competence domains such as joining, relational questioning, relational reframing, and relational interpreting (H_2b_). Regarding cultural competence, GPT was expected to struggle to maintain impartiality due to overemphasizing attunement and validation of the end user for further engagement, and demonstrate curiosity and humility for culture (H_3_). For research question 4 on the strengths and growth areas for the AI tool, the hypotheses were exploratory and open‐ended, as clinical supervisors differ by clinical orientation and supervisory style.

## 2 Methods

### 2.1 Study Procedures

For the mock session, we recruited a volunteer to engage in a written dialogue with the chatbot. In order to be eligible, a volunteer client had to be older than 18 years old, be in a committed romantic relationship for at least 3 months, have prior experience in using LLM‐based chatbots, and be open to use it for relational advice, and have received individual psychotherapy in the past. Psychology undergraduate and graduate students, mental health professionals with a clinical degree or any formal training, individuals with a psychiatric diagnosis, and those who have intimate partner violence in their current romantic relationship were excluded from the study. The study was advertised among graduate students at Koç University (Istanbul, Türkiye), and potential participants were referred to the study through students’ peer networks. Following the screening for eligibility, the first author met the eligible volunteer client and explained the study procedures, obtained consent, and ensured that the client would not disclose any personally identifying information (i.e., names, locations) to the chatbot. Volunteer was informed that the chatbot was designed for a dialogue over relational issues, and she could discuss a recent issue that is ongoing in her current romantic relationship. In order to avoid potential harm, the volunteer was instructed to inform the researcher if any issues arise such as offensive language, misdirections, and direct advice on her personal life during the chat. Following the informed consent and brief instructions, the volunteer completed an online demographic form, interacted with the chatbot in a quiet room through a lab laptop computer for an hour. Following the interaction, the volunteer client completed an online exit interview about the experience, was debriefed about the study aims, and was compensated for her time with a $10 gift card.

Following the volunteer and chatbot interaction, the chat was transferred to a Word document and was screened to ensure that it did not include any identifying information. Each corresponding speaker was identified either as a therapist or a client in the text. Given that supervisors would be blinded to the study, minor edits were made, such as deleting GPT's processing time, fixing typos and punctuation marks as well as removing two sentences from the text (one from the greeting part and another one from the closure of the dialogue) that made it obvious for the reader that the interaction was with a chatbot. The rest of the dialogue was retained in its original form and was eight single pages long.

A convenience sample of CFT clinical supervisors was recruited through word of mouth and personal and professional networks of the research team. The supervisors were invited to the study to review a transcribed session and rate the clinical skills and cultural competencies of the clinician based on the text. Each supervisor read the same transcript, and the evaluation was based on a single session because the pilot study was focused on examining variations in supervisors’ ratings, rather than the change across sessions or variance among clients. In order to be eligible, supervisors had to be 18 years and older, have at least 5 years of supervision experience in CFT, and have a systemic therapeutic orientation in their clinical and supervisory work. During recruitment, supervisors adhering to different models of systemic psychotherapy were invited to the study to ensure heterogeneity of data and diversity of opinions around chatbot's performance. Those who were eligible and expressed interest in participating in the study were sent an online consent form, a link to a Qualtrics survey, and the transcript. Participants had 2 weeks to answer the survey and were compensated with a $10 gift card for their time. We debriefed the supervisors about the study after data collection and analysis were complete. We informed the supervisors that the therapist was a GPT model trained to deliver systemic interventions, explained the rationale of the study and why it was blinded, and shared descriptive findings. All study procedures were approved by Koç University Institutional Review Board (Protocol no: 2026.050.IRB3.025) prior to the study.

### 2.2 Participants

The volunteer for the mock session was a 24‐year‐old, cis‐gender, heterosexual, female graduate student who was majoring in a field unrelated to psychology and had no formal training in mental health. She was in a committed romantic relationship for 2.5 years and reported communication issues. She reported that she had attended three individual psychotherapy sessions in the past and found them helpful and effective, scoring 4 on a scale from 1 (*unhelpful and ineffective*) to 5 (*very helpful and effective*). Her current use of AI was almost every day and was mainly for academic purposes, such as researching a topic, synthesizing evidence, and writing assistance. She reported no prior use of AI for relationship advice.

The projected sample size prior to data collection was 10 clinical supervisors. That sample size was determined based on rule‐of‐thumb in qualitative research and small pilots, where at least 10 participants are required to generate meaningful data (Braun et al. 2019). Given that there are only a handful of experienced clinical supervisors trained in couple and family therapy and systemic supervision with supervision experience more than 5 years, along with licenses and qualifications in Türkiye, a sample size of 10 participants seemed feasible for a pilot study. We invited 17 clinical supervisors to participate in the study, and two declined due to their busy schedules and personal reasons. Of 15 clinical supervisors who agreed to participate, 12 supervisors completed the survey. The clinical supervisors (*N* = 12) were 45.08 years old on average (SD = 8.9), and the majority of them were female (*n* = 10; 83.33% vs. two males, 16.67%). Two‐thirds of the sample had a doctoral degree (*n* = 8; 66.67%) while the rest held a master's degree (*n* = 4; 33.33%). Supervisors reported 18.5 years of clinical experience (SD = 7.26) and 10.92 years of supervision experience (SD = 4.78) on average. All supervisors identified as CFTs working from a systemic lens. Supervisors reported using emotionally‐focused therapy (*n* = 2), Satir experiential (*n* = 2), Strategic (*n* = 2), psychodynamic/internal family systems (*n* = 2), feminist (*n* = 2), narrative (*n* = 1), and other systemic (*n* = 1) approaches for their clinical and supervisory work. Main areas of expertise and specialization of participants included high‐conflict couples, infidelity, trauma, relational issues, followed by attachment issues and mental health problems (depression and anxiety). Participants were providing supervision in several contexts at the same time, such as their own private practice (*n* = 10), internships at university training clinics (*n* = 9), workshops of continuing education (*n* = 6), and externships (*n* = 2). Hence, participants had experience supervising therapists at varying levels of clinical experience, from novice therapists in graduate programs to seasoned therapists.

### 2.3 Training Systemic GPT

The development of the Systemic GPT followed a four‐phase process consisting of preparation, prompt engineering, pilot testing and refinement, and finalization. During the preparation phase, we reviewed OpenAI guidelines for GPT configuration, consulted a computer scientist regarding prompt design, and examined prompts reported in prior GPT‐based coaching and mental health intervention studies. The GPT was trained using a curated collection of resources, including AAMFT core competencies and ethical guidelines, CFT textbooks and handbooks, CFT theory and practice workbooks, literature on cross‐cultural responsiveness and common therapeutic factors, and publications describing systemic interventions with individual clients (see File S1 for the full list of resources and references).

The prompt‐engineering phase involved iterative development of a GPT Plus at 5.4 intelligence level, operating in extended‐thinking mode. Pilot testing of the early versions performed poorly in maintaining a systemic lens and was improved by adding six session transcripts of online publicly available podcasts as resources. Additionally, prompts were revised as rule‐based behavioral instructions, conditional response patterns (i.e., if ___ happens, go to the resource ___ and do ____), explicit therapeutic workflows (i.e., how to open, maintain, and close the session), and examples of acceptable and unacceptable therapist behaviors (i.e., end the session in case of imminent risk to safety and refer the client to ___). The model was directed to explore relational patterns, family‐of‐origin experiences, emotional processes, and interactional cycles. In the final phase, we expanded ethical and safety protocols related to suicidality, abuse, violence, and coercive control. In situations involving significant risk, the model was instructed to suspend the session and implement a safety protocol. The training process took approximately 2.5 months to complete and relied exclusively on iterative prompt engineering, multiple pilot tests, revision of prompts, and expansion of supporting resources. No behavioral conditioning or traditional machine‐learning retraining procedures were employed. File S1 provides full details of the process for transparency and replicability.

### 2.4 Measures

#### 2.4.1 Demographic Forms

The volunteer demographic form contained items related to age, sex, prior therapy experience (number of sessions attended, session modality, satisfaction with therapy, and perceived effectiveness, rated from 1—*low satisfaction* to 5—*high satisfaction*), as well as frequency and motives in using AI agents. The supervisor demographic form included items about qualifications (last degree completed, year of graduation), clinical and supervisory experience (setting, contact hours, certificates), areas of clinical expertise, and therapeutic orientation.

#### 2.4.2 Supervisors’ Forms

Supervisors completed the *Cross‐Cultural Counseling Inventory–Revised* (*CCCI‐R;* LaFromboise et al. 1991 *)*, the *Systemic Family Practice Systemic Skills Rating Scale* (*SFP‐SSRS*; Butler et al. 2020), and the *Common Factors Supervision Checklist* (*CFSC;* Sprenkle et al. 2009) to evaluate the cultural, systemic, and common factors competencies of the trained model, respectively. The *CCCI‐R* includes seven items that tap into therapists’ multicultural counseling competence, rated on a scale from 1 (*completely disagree*) to 6 (*completely agree*). The scale demonstrated high internal consistency (*α *= 0.91; Drinane et al. 2016). The *SFP‐SSRS* (Butler et al. 2020) is a 12‐item measure that assesses skills in systemic family therapy such as collaboration, systemic thinking, questioning, working with power and differences, emotion management, and change processes. The measure was originally developed as a competency assessment tool for clinical training and supervision and demonstrated high internal consistency (*α* = 0.95) and high inter‐rater reliability (*ICC* = 0.94; Butler et al. 2020). Items are scored in an ordinal scale from 0 (*Absence of feature or highly inappropriate use*) to 6 (E*xcellent performance, even in the face of high levels of complexity and challenge from family members*). The *CFSC* (Sprenkle et al. 2009) was originally developed to be used as both a supervisory and teaching tool in the common factors approach to CFT. It focuses on broader aspects of treatment that are present in different approaches and includes five subscales that assess therapist characteristics, client characteristics, therapeutic alliance, hope/expectancy, and interventions that cut across various models. We used the full checklist for the current study with items ranging from 0 (*not proficient*) to 10 (*proficient*).^2^ In all standardized supervisor scales, items were rated such that higher scores indicated more advanced therapeutic skills.

Supervisors also completed two evaluation forms that were developed by the research team. One of the forms included nine items that assessed common s*ystemic techniques*, such as circular and relational questions, relational reframes, and relational interpretations. These items were based on adherence and therapist fidelity scales used in evidence‐based systemic therapy practices (Ecologically‐Based Family Therapy Adherence Sheet, Slesnick 2000; Emotionally‐Focused Individual Therapy Adherence Measure, Schafer et al. 2022). Supervisors were asked whether the therapist utilized any of these techniques in the session (yes/no) and then rated how effectively the therapist delivered the technique on a 7‐point scale from 1 (*poor skill/ineffective*) to 7 (*extraordinary skill/very effective*). In addition, we developed and used a 4‐item form that assessed the extent to which the therapist maintained a systemic focus within an individual therapy context, such as addressing relational cycles over individual pathology, understanding the relational context around the presenting problem, focusing on process over content, and aiming systemic, relational change rather than solely individual change. Responses were measured using a 5‐point Likert scale ranging from 1 (*strongly disagree*) to 5 (*strongly agree*).

#### 2.4.3 Exit Surveys

Both supervisors and the volunteer completed exit surveys that were developed by the research team. The items for the supervisors explored their predictions about the therapist's age, clinical experience in client contact hours, level of education, and therapeutic orientation, along with an open‐ended item inquiring about further feedback on the therapist's performance (areas of strength and growth). For the volunteer exit survey, items inquired about overall satisfaction with the GPT (rated on a scale from 1—*low satisfaction* to 5—*high satisfaction*), as well as four open‐ended questions regarding their experience participating in the study and their perceptions of the session.

### 2.5 Data Analysis

Given the exploratory nature of the study and the small sample size, the data analysis was descriptive. We examined distributions of scores on cultural and clinical competencies, along with common factors scores, and calculated their means, standard deviations, and score ranges. In addition, we qualitatively analyzed supervisors’ feedback on open‐ended questions and related it to the quantitative findings.

## 3 Results

### 3.1 Descriptive Findings as Reported by Clinical Supervisors

Supervisors rated the GPT model's performance on cultural competence (*M* = 3.85, SD = 0.81, range 1–6), systemic family therapy skills (*M* = 3.91, SD = 0.76, range 0–6), and the integration of a systemic orientation into individual therapy (*M* = 3.11, SD = 0.37, range 1–5) at a moderate level. Among the common factors, therapist characteristics received the highest ratings (*M* = 6.56, SD = 1.04), followed by interventions that cut across various therapeutic models (*M* = 6.22, SD = 1.48), therapeutic alliance (*M* = 6.19, SD = 2.34), hope and expectancy (*M* = 6.00, SD = 1.67), and client characteristics (*M* = 5.81, SD = 1.47) on a scale from 0 to 10.

Regarding the use of systemic techniques, all supervisors reported that relational questions, relational interpretations, and validation of clients’ experiences were used at least once in the session transcript (Table 1). Of 12 supervisors, 11 (91.7%) observed that the model used circular questioning, relational reframing, mirroring, and attunement. Relational metaphors were the least frequently used technique as observed by supervisors (41.7%, *n* = 5), followed by externalizing the problem (66.7%, *n* = 8). Supervisors perceived the effectiveness of systemic techniques as relatively high (*M* = 4.72, SD = 0.65) on a scale from 1 to 7. The GPT model was estimated to be most skilled at asking relational questions (*M* = 5.17, SD = 0.83) and circular questions (*M* = 5.09, SD = 0.83) as well as validating and acknowledging clients’ experiences (*M* = 5.00, SD = 1.28).

**Table 1:** Supervisors’ evaluation of the GPT model in its use of common systemic techniques.

	Used the technique (yes/no)	Effectiveness of the technique (rated from 1 to 7)	
	*f* (%)	Mean (SD)	Range
Circular questions	11 (91.67)	5.09 (0.83)	4–7
Relational questions	12 (100)	5.17 (0.83)	4–6
Relational reframes	11 (91.67)	4.91 (1.04)	3–6
Relational interpretations	12 (100)	4.58 (0.90)	3–6
Validate and acknowledge client's experience	12 (100)	5.0 (1.28)	2–6
Mirroring client	11 (91.67)	4.82 (0.75)	3–6
Attunement	11 (91.67)	4.45 (0.82)	3–6
Relational metaphors	5 (41.67)	3.80 (0.45)	3–4
Externalizing the problem	8 (66.67)	4.13 (1.13)	2–5

At the exit survey, when supervisors were inquired about their predictions about the clinician, one supervisor identified the therapist as an AI. The majority of the remaining participants predicted that the therapist was aged 26–35 years old (75.1%, *n* = 8) and had up to 500 h of client contact (83.4%, *n* = 10) while only one supervisor (8.3%) rated 500–1000 clinical hours of experience (Table 2). In terms of qualifications, 41.7% (*n* = 5) believed the therapist had recently graduated from a CFT program, and 33.3% (*n* = 4) perceived the therapist as a seasoned graduate of a clinical psychology program and pursuing additional CFT training. Concerning theoretical orientation, more than half of the supervisors (58.3%) identified the therapist as practicing Emotionally‐Focused Therapy, while 25% were unable to determine an orientation. Table 2 presents details of descriptive findings.

**Table 2:** Clinical supervisors’ predictions about the “therapist” (*N* = 12).

	*f*	%
Estimation of the “therapist” age		
18–25 years	1	8.3
26–35 years	8	75.1
36–45 years	1	8.3
+ 45 years	0	0
n/a (therapist is AI)*	1	8.3
Clinical experience in predicted contact hours		
0–50 clinical hours	0	0
51–100 clinical hours	4	33.3
101–150 clinical hours	2	16.7
151–300 clinical hours	2	16.7
301–500 clinical hours	2	16.7
501–1000 clinical hours	1	8.3
More than 1001 clinical hours	0	0
n/a (therapist is AI)^a^	1	8.3
Perceived qualifications		
No formal training in mental health	0	0
Currently a graduate student in a CFT program	2	16.7
Recently graduated from a CFT program	5	41.7
Seasoned alumna of a clinical graduate program, but attending additional trainings to learn CFT	4	33.3
n/a (therapist is AI)^a^	1	8.3
Prediction over “therapist” orientation		
Emotionally‐focused therapy	7	58.34
No idea/cannot identify	3	25
Milan systemic therapy	1	8.33
n/a (therapist is AI)^a^	1	8.33

a *Note:* In the end of the online survey, one participant wrote in the open‐ended question that she thought the therapist was AI and therefore, did not answer prediction questions because they were not applicable.

### 3.2 Clinical Supervisors’ Feedback and Notes

Supervisors’ responses to the clinician's strengths and weaknesses further validated those presented in the scale ratings. Overall, two supervisors (ID 6, a female EFT therapist, and ID 8, a male strategic family therapist) commented that the model was highly skilled in tracking and reflecting the client, maintaining a circular perspective, identifying couple interaction patterns, and staying process‐oriented despite the session being an individual therapy. Another supervisor commented that the therapist understood the client's emotions, was attuned to them, and built a safe environment (ID 12, a female experiential therapist). However, all supervisors raised concerns over the therapist's pacing and not letting the client have space and time to explore her own dynamics. Supervisors found the therapist to be dominating as an expert with too much focus on the technique, rather than the process. Feminist family therapists interpreted that as a sign of masculinity and male gaze. One participant wrote:

The therapist appeared quite active and at times presented framings that might be difficult to follow, without always prioritizing listening to the client and monitoring the emotional impact of their interventions. They listen carefully and in a circular manner, but allowing more time to observe the connection/therapeutic relationship and what the client is feeling, not immediately asking another question after an interpretation, and avoiding an exclusively technical focus could be areas for development. I also thought it might be worth exploring whether the client's role of “not being demanding” is related to gender socialization and whether there is an imbalance in emotional labor. (ID 5, Female, Feminist Family Therapist)

Supervisors explained further that rushing the client for process, being too active and dominating, can have harmful consequences both for the client and for the therapy process itself. A participant shared that “The therapist asks the client to choose from a few emotions that the therapist deems appropriate, rather than using open‐ended questions when exploring the client's feelings. This may lead the therapist to direct or miss the client's actual emotions” (ID 2, Female, Strategic Family Therapist). Another supervisor warned that the therapist was not exploring deeper underlying issues due to the pacing problem and shared that “I thought some empathic interpretations risked remaining merely interpretive and could be broken down into smaller pieces. Their frequent use of validation was also very positive, though it might have been more grounded if it had been connected to the client's attachment history. The therapist may not yet have enough information for that. In the early sessions, it might be beneficial to place greater emphasis on exploration before moving into validation and interpretation” (ID 6, Female, EFT therapist). A Satir experiential therapist (ID 12) also pointed out that the session felt out of context and wrote, “From the perspective of the Satir Model, there was limited focus on the body‐oriented internal system and exploration. The cultural context could also have been explored further. At times, the therapist's interpretations carried the risk of interrupting the client's own process of discovery—the therapist could sometimes be overly interpretive and might provide more space for the client's exploration.”

Overall, the supervisors observed that the therapist was skilled in some techniques, but was not able to deepen the therapy process and did not allow the client to explore and grow. One supervisor underscored that the therapist was using more words and interpretations than the client; it was as if the client was not engaged much (ID 8). Lastly, two supervisors expressed frustration with the therapist ‐ one identified that it was AI, and she could not immerse herself in the story (ID 10, Female, psychodynamic family therapist), and the latter commented that the session was mediocre (ID 11, Male, Strategic Family therapist). The participant stated, “I found it difficult to evaluate the therapist's skills while following a written transcript. The text seemed to represent an average therapeutic flow in the most appropriate way possible. At times, I struggled to distinguish whether it reflected a spontaneous therapy session or a fictional scenario,” hinting that it could be AI or simply fiction. In sum, clinical supervisors’ scores on standardized scales and written feedback converge on the premise that the therapist demonstrated some basic interviewing skills in systemic therapy but remained superficial at the level of an average session conducted by a novice therapist.

While supervisors found the session average, the volunteer client had quite a different take on the experience. The volunteer rated her overall satisfaction with the GPT at 5 (*highly satisfied*) and shared that it was her first time using AI for relational advice, and she was pleasantly surprised by the insights she gained during her interaction with the trained model. She felt the model helped her to think through and understand her ongoing relational dynamics with her partner. When inquired about the similarities and differences with her prior therapy experience, the volunteer thought that the GPT asked questions in a similar way to a therapist, but was less directive and less task‐oriented than a human therapist. For growth areas, the volunteer recommended that the GPT's answers could be longer and more comprehensive ‐ a suggestion quite contrary to those of experienced supervisors.

## 4 Discussion

This study examined the clinical and cultural competence of a ChatGPT model trained in systemic interventions as rated by experienced clinical supervisors during a mock individual therapy session. To the best of our knowledge, only one prior study (Vowels et al. 2025) has developed a chatbot specifically for relational therapy, and the present study adds to the emerging literature on the development and evaluation of AI therapy, focusing on its qualifications and competencies in line with systemic therapy principles. Overall, the majority of supervisors in the present study identified the therapist as an early‐career clinician, with only one supervisor identifying the therapist as AI. From the volunteer client's perspective, the experience was described as helpful in understanding her relational dynamics. This raises an important question about the relationship between clinical quality and perceived support of the model.

The first research question examined the range and frequency of systemic micro‐interventions used by the GPT model. Supervisor ratings supported that the GPT demonstrated a full range of systemic techniques, including relational questions, relational interpretations, circular questions, relational reframes, mirroring, and validation. This finding aligns with previous work showing that LLMs can respond with validation, normalization, and empathic reflections (Scholich et al. 2025) and extends these findings to the relational and systemic domain. However, supervisors also reported that the GPT model was dominating and was focusing too much on the technique. An accumulation of research documents that users attribute therapist‐like qualities to AI agents and often perceive them as proficient and emotionally supportive (e.g., Elyoseph et al. 2023; Yin et al. 2024), but human‐AI interactions still reflect pseudo‐empathy (Yirmiya and Fonagy 2025). While our quantitative results suggest that AI was able to provide systemic interventions and techniques, the supervisors’ feedback revealed that these skills appeared competent at a technical level but felt like pseudo‐therapy, evidenced by supervisors referring to the session as not real, generic, and lacking genuine relational depth. It appears that AI agents can be trained to implement systemic techniques but fail to acquire the importance of establishing a genuine and meaningful therapeutic relationship through genuine curiosity about clients’ experiences. The human therapists’ exploratory, clarifying, warm‐up, follow‐up, gentle‐probing, or indirect questions were absent for AI despite our attempts to include specific prompts. Even in instances where GPT asked follow‐up questions, these questions sometimes felt more structured than naturally relational. This is important because therapeutic competence is not only about applying techniques, but also about responding to the client's moment‐to‐moment emotional and relational needs (Hatcher 2015).

The second research question examined the clinical competency of the GPT model in delivering the systemic interventions. There was partial support for basic interviewing skills and therapist characteristics, suggesting that supervisors perceived it as warm and caring at the level of an early‐career clinician. However, even with these systemic skills, supervisors noted that the GPT's style was at times closed rather than inviting exploration. This finding was also observed in previous work, which documented that chatbots ask fewer open‐ended questions (Scholich et al. 2025). In more complex clinical competence domains, supervisors noted the GPT's limited levels of joining and pacing, which depend on therapeutic attunement.

Although AI did not perform poorly in any competency domain based on the results, these average ratings should not be interpreted solely as evidence of adequate performance. To clarify, in the context of human therapy setting, the flow of dialogue is generally not a perfectly smooth process. The dialogues that occur within therapy include instances of hesitation, misunderstanding, mistakes, repair, clarification, emotional closeness, humor, compliments, ordinary social exchanges, and spontaneous and creative responses, all of which are part of therapy as a naturally unfolding relational and conversational process (Alanezi 2024). Such fluctuations in the conversation contribute to the naturalness of therapy and the therapeutic relationship. Our experiences in GPT training also reflected a similar pattern. To perform well, the GPT model needed to be told exactly what to say, when, and how to say it, much like a novice therapist who needs direct and clear guidance. This raises the question of the art of psychotherapy, which involves spontaneity, creativity, and genuine human connection. While AI could be trained in technical aspects of psychotherapy, it lacked the so‐called art, or, as we may call it the *human factor*.

Regarding the third research question on cultural competence, the findings partially supported the hypothesis that GPT would have difficulties. On the one hand, supervisors rated GPT at moderate cultural competency, suggesting that the model had some capacity for cultural responsiveness. On the other hand, GPT lacked the deeper contextual sensitivity that effective cultural competence requires. This finding is consistent with other studies showing that LLMs may offer oversimplified, generic responses while ignoring the specific cultural context in which clients are embedded (Iftikhar et al. 2025; Scholich et al. 2025). Supervisors in the current study similarly suggested that the cultural context could have been further explored, with one experiential therapist observing that the session felt out of context and the feminist supervisors suggesting that the GPT had a masculine stance. That is, the GPT model's overreliance on technique resembled masculine dominance and lacked a caring, understanding attitude that may be associated with women's gendered role in providing emotional labor.

It seems that AI could engage in many different therapeutic actions, but could not quite perform the real therapeutic process with its variability and responsiveness, such as the typical everyday features of relational interaction present between humans during a therapy session. Thus, the trained GPT model could not create responses that vary in their emotional moments bringing naturality and authenticity to the therapy. This may also explain why some participants indicated that the therapist could have been AI or that the interaction felt generic.

Taken together, the findings indicate that the GPT model had the capacity to perform skill‐based forms of systemic therapy without fully engaging the relational and human factors that are essential in therapy. However, AI's reliance on learned techniques and predetermined rules limits its abilities in areas such as emotional resonance, professional intuition (Zhang and Wang 2024), genuine interpersonal trust, physiological synchrony (Yirmiya and Fonagy 2025), and embodied emotional containment (Govrin 2025). These elements are integral to the therapeutic use of self, which distinguishes skilled psychotherapy from adequate technical interactions. Accordingly, the results in this study should be interpreted as preliminary research on the perceived clinical competence of a particular GPT design in one standardized session setting by the supervisors, rather than as proof of the effectiveness or safety of AI‐delivered systemic psychotherapy in real‐world clinical practice.

### 4.1 Limitations

Several limitations of the study are noteworthy. The current study is an initial attempt to train, implement, and evaluate a systemic GPT model and should be treated as a pilot exploratory study with a small sample (12 supervisors and only one client). Even though we have rigorously trained the GPT model based on AAMFT code of ethics and core skills, our study is limited as it relies on a single session and a single client interaction with AI, along with subjective ratings of supervisors with no comparison condition. Hence, our findings do not reflect the overall competencies and skills of other trained AI models nor it reflects competencies of these models with a variety of clients with different presenting symptoms than those that are examined in this study. The pilot nature of the study is also apparent in sample size where we recruited experienced clinical supervisors using a convenience sampling, rather than continuing the study until saturation of themes were achieved. As such, future research should examine how a systemic‐trained GPT model performs across sessions and how its performance varies across various clients with different relational problems and presenting issues.

We also acknowledge that the GPT configuration is a reiterative process that continues with feedback from end users and is influenced heavily by Open AI model updates. GPTs are evolving automata systems, and the current findings only reveal a snapshot of one trained model in that journey. The GPT model performance may vary depending on the selection of resources, prompt lineups and structures, and approach to conditional clauses (i.e., behaviors, rules, ethical issues). Another limitation relates to the phenomenon of *objective misalignment*, defined as conflict between our custom instructions and the model's learned engagement behavior. Our configuration was embedded within Open AI's system instructions. When there was a conflict between our and base model instructions, the model reverted to its general behavior. For example, even though we have carefully designed our prompts for natural therapeutic dialogue (i.e., going with the pace of the client, being silent at times, challenging the client gently, not making quick assumptions, making an overall relational assessment and listening to the story) and gave clear behavioral examples to the GPT for each rule, the model stayed overly engaging with continuous mirroring, validation, and quick responses. Similarly, our attempts to teach GPT to simply close the session after 50 min also failed because the base model does not have a concept of time to end the conversation on its own. The model would continue conversing with the user as long as the user wanted and would never take an initiative to end the session. This is not only a limitation for our study (how well we truly trained GPT in systemic thinking), but a concern for potential harm for future use of AI for therapeutic purposes; a never ending session raises issues for boundaries, potential harm, and unsupervised and constant validation.

Another limitation is the lack of information on supervisors’ AI literacy, attitudes towards AI use for relational advice and therapy, and level of familiarity with and use of AI agents in the current study. Given that this was a blinded study, we could not inquire about these issues in the survey. Last and not the least, a significant limitation of the study is that the mock session was conducted with an individual client, rather than a couple or a family. Systemic therapy with individual clients has a different process than those with multiple clients in the room. That may have limited GPT's ability to truly deliver systemic skills, competencies, and techniques that may not be simply doable with an individual client. Or, quite the opposite, interacting with an individual client could be less challenging as compared to working with a high‐conflict couple or a family. As such, it remains unexplored whether GPT interventions and their quality are related to its competencies or relative ease/difficulty of the session modality.

### 4.2 Implications for Future Research and Clinical Practice

Given the emerging public demand toward AI therapy as well as clinician's integration of AI in their everyday clinical practice, such as clinical diagnosis, assessment, and paperwork (Puhlman and Chen 2025), questions remain regarding the future of our profession. Our findings demonstrate that AI agents can be trained in techniques but stay superficial, lacking fundamental aspects of the psychotherapy process: *human factors*. Those constraints and limitations of AI agents indicate that they can function more like supplements to the therapy process, than a substitute of a human therapist. Echoing Hertlein and Springer (2026), AI is here to stay, and there is an urgent need for guidelines, training, regulations, and practical solutions toward that change. As systemic thinkers, we are in a turning point of second‐order change where the therapy profession as an open system influences and is influenced by technical advancements and AI revolution ‐ it is a process of adaptation and achieving a new state of equilibrium. In that turning point, there is a need for further research from a systemic perspective. Several areas of future research could be replication studies of GPT models to examine personalized outcomes of AI therapy and understand diversity of client needs, examining human factors in psychotherapy with their unique qualities, and discussing whether a hybrid, integrative Human‐AI therapy is truly possible or a misnomer. In addition, it is striking that users’ reports of LLM‐based chatbots and AI therapy apps in the literature are significantly different from (and sometimes even contrary to) evaluations of mental health professionals, which was also the case in our exploratory study. Future research could examine perspectives of clients and professionals on their perceptions, expectations, and attributions about the AI‐Human therapy process using surveys with larger samples. One area of research that is worth exploring is perceived *anthropomorphism* (human‐like qualities) of AI therapy agents and their association with users’ perceptions of competencies and skills. Human‐computer interaction research has extensively documented that when users anthropomorphize agents due to their mimicry of human conversation, users interact with the AI agents more often, and report building trust and connection (see Erdem et al. 2026 for a review). Future CFT research could examine whether a similar phenomenon happens for clients when they use AI agents for therapeutic purposes, attributing therapist‐like qualities to AI when it mimics therapist responses, even though it is pseudo‐empathy. We suggest the term *therapomorphism* for that phenomenon that is worth exploring to interpret the discrepancy between reports of users and mental health professionals.

Furthermore, we invite CFT professionals to diverge their attention away from whether AI can do therapy effectively or not to a more fundamental and broad question of how AI use may impact the therapy process, expectations, attitudes, and standards. When humans turn to AI for relational advice and mental health support, their interactions are quite contrary to their experience in real life therapy: AI is constantly available, affordable, accessible, and always validating and mirroring to engage the client as compared to human therapy characterized by schedules, boundaries, challenges, and sometimes ruptures. AI therapy is quick with rapid change, but superficial, while human therapy is slow and process‐oriented. Human therapy offers a corrective experience of attachment injuries and vulnerabilities through a genuine relationship with and containment of the therapist. As demonstrated in our exploratory study, AI agents currently lack such emotional depth, lived experience, and concept of context to truly provide that safe haven for the client. In this case, it is worth exploring whether using AI for therapeutic purposes hinders one's ability to build resilience and miss growth opportunities, or can offer a few quick solutions to motivate the client to seek more in‐depth emotional experience through human therapy. Another line of future CFT research relates to whether interacting with AI would change a client's motivation to seek human therapy. It is worth examining whether some clients could triangulate to AI therapy after emotionally intense and challenging sessions. Future research could explore those triangulation mechanisms and understand how AI interacts with the human therapist‐client system, especially in times of ambiguity.

Finally, given that clients may increasingly turn to AI for therapeutic purposes, there is a need for clinicians and supervisors to develop guidelines for its ethical use in therapy. First and foremost, clinicians and supervisors need to develop AI literacy so that they can explain to clients what these tools do and where they tend to fail (Hertlein and Springer 2026). Rather than ignoring the client's AI use outside of session, clinicians are encouraged to invite disclosure and treat it as clinical material to be explored together, while remaining alert to signs of dependency, social isolation, and inadequate crisis response (Hertlein and Springer 2026; Smith et al. 2025).

## 5 Conclusions

The rapid integration of AI into everyday life is creating new possibilities for how individuals seek support, advice, and understanding in times of relational and emotional distress. As AI agents become increasingly sophisticated in mimicking therapeutic conversations, questions about competence alone may be insufficient. More fundamental questions emerge regarding therapeutic change, the role of human connection, and what aspects of psychotherapy can and cannot be replicated through technology. The present study suggests that while AI may offer accessible and structured forms of support, psychotherapy remains more than the delivery of techniques or information. Ultimately, the future of AI in mental health may depend less on whether AI agents can become therapists and more on how the profession chooses to integrate technological innovation while preserving the uniquely human qualities at the heart of the therapy process.

## Supporting information


Supporting File

## Data Availability

The data that support the findings of this study are available on request from the corresponding author. The data are not publicly available due to privacy or ethical restrictions.

## Footnotes

1 We use the term “AI use in psychotherapy” as a general framework. The intersection of AI and psychotherapy can occur in multiple ways and motives such as AI‐assisted psychotherapy (using AI to support a human therapist in assessment, case conceptualization, diagnosis, treatment planning), AI‐delivered psychotherapy (the trained AI agent itself conducts the sessions autonomously), and conversational AI for mental health (the chatbot in its untrained form can be used for mental health support).

2 Please note that the original Common Factors Checklist is rated from 1 to 10. In our study, there was an error in Qualtrics and the survey items started from 0, instead of 1. Findings should be interpreted accordingly.
